# Gliosis attenuation in experimental autoimmune encephalomyelitis by a combination of dimethyl fumarate and pregabalin

**DOI:** 10.3389/fncel.2022.921916

**Published:** 2022-08-12

**Authors:** Amanda Garcia Hoelz, Danielle Bernardes, Luciana Politti Cartarozzi, Alexandre Leite Rodrigues de Oliveira

**Affiliations:** Department of Structural and Functional Biology, Institute of Biology, University of Campinas, Campinas, Brazil

**Keywords:** experimental autoimmune encephalomyelitis (EAE), microglia, astrocytes, dimethyl fumarate (DMF), pregabalin (PGB)

## Abstract

Dysregulated microglia and astrocytes have been associated with progressive neurodegeneration in multiple sclerosis (MS), highlighting the need for strategies that additionally target intrinsic inflammation in the central nervous system (CNS). The objective of the present study was to investigate the glial response in experimental autoimmune encephalomyelitis (EAE)-induced mice treated with a combination of dimethyl fumarate (DMF) and pregabalin (PGB). For that, 28 C57BL/6J mice were randomly assigned to the five experimental groups: naïve, EAE, EAE-DMF, EAE-PGB, and EAE-DMF + PGB. Pharmacological treatments were initiated with the beginning of clinical signs, and all animals were euthanized at 28 dpi for the lumbar spinal cord evaluation. The results demonstrated a stronger attenuation of the clinical presentation by the combined approach. DMF alone promoted the downregulation of Iba-1 (microglia/macrophages marker) in the ventral horn compared with the non-treated EAE animals (*P* < 0.05). PGB treatment was associated with reduced Iba-1 immunofluorescence in both the dorsal (*P* < 0.05) and ventral horn (*P* < 0.05) compared to EAE vehicle-treated counterparts. However, the combined approach reduced the Iba-1 marker in the dorsal (*P* < 0.05) and ventral (*P* < 0.01) horns compared to non-treated EAE animals and further reduced Iba-1 in the ventral horn compared to each drug-alone approach (*P* < 0.05). In addition, the combination of DMF and PGB reduced activated astrocytes (GFAP) in both the dorsal and ventral horns of the spinal cord to a naïve-like level and upregulated Nrf-2 expression. Taken together, the data herein suggest robust attenuation of the glial response in EAE mice treated with DMF and PGB.

## Introduction

Multiple sclerosis (MS) is the most prevalent neurodegenerative disease afflicting young adults with 2.8 million cases around the world (MSIF, 2020). The main theoretical hypothesis for MS development points out to an initial activation and infiltration of immune cells from the periphery to the central nervous system (CNS) leading to neuroinflammation, demyelination, gliosis, and neurodegeneration (Ransohoff et al., 2015). Therefore, most of the current disease-modifying therapies (DMTs) target the recruitment and capture of cells from blood to the CNS intending to inhibit the cascade of events that eventually lead to the neurodegenerative component (Dargahi et al., 2017). Although great progress has been made in MS therapies by highly improving the quality of life of the affected individuals, the disease progression is still an imperative debate (Gholamzad et al., 2019). Notably, it has been suggested that the inflammatory component within the CNS, i.e., microglia and astrocytes, may not be significantly influenced by the peripheral immune cells at chronic phases of the disease, so they are essentially linked to the progressive neurodegeneration observed in MS patients (Dendrou et al., 2015; Correale et al., 2019).

Indeed, important bidirectional crosstalk between astrocytes and microglia has been suggested. On one hand, it has been considered that microglia can secret growth factors and inflammatory molecules that modulate astrocyte function to a neurotoxic activity that is harmful in several neurodegenerative diseases, including MS (Greenhalgh et al., 2020). On the other hand, it has been argued that astrocytes may release several activating factors as well as adhesion molecules and chemokines that can mediate additional attraction of microglia and other immune cells to the lesion sites, which may disseminate the neurodegeneration (Correale et al., 2019; Aharoni et al., 2021). Therefore, combined approaches targeting peripheral immune cell function but also the CNS-intrinsic inflammation have been suggested as they could promote a more efficient chronic neuroprotection in MS (Dendrou et al., 2015).

Dimethyl fumarate (DMF) has been approved in the last decade in several countries as a truly promising strategy (Mills et al., 2018; Gholamzad et al., 2019). A long-term study of the effects of DMF on MS patients has suggested reduced disability progression as well as improved cognition with reduced events of depression and fatigue (Salter et al., 2021). Some studies have suggested that DMF metabolite, monomethyl fumarate, can cross the blood-brain barrier and thereby promote antioxidative and neuroprotective effects (Moharregh-Khiabani et al., 2009; Linker et al., 2011). *In vitro* studies have even demonstrated a reduced inflammatory as well as oxidative profile in LPS-activated microglia and astrocytes by DMF administration (Wilms et al., 2010; Lin et al., 2011; Parodi et al., 2015; Michell-Robinson et al., 2016). However, in MS animal models such as experimental autoimmune encephalomyelitis (EAE), some conflicting results have been described. For example, DMF treatment promoted improvement of cognitive function which was associated with reduced astrocytic but increased microglial reactivity in the fimbria of EAE animals (das Neves et al., 2020). Therefore, it has been suggested a combination of DMF and other pharmacological strategies to reach more successful results (Lian et al., 2018; Pouzol et al., 2019).

Indeed, DMF alone may not improve all MS-related symptoms which require a combination of pharmacological treatments such as pregabalin (PGB). The PGB mechanism of action is the blockage of presynaptic voltage-sensitive calcium channels decreasing the neurotransmitter release, which is associated with diminished epileptic seizures, but also with attenuation of neuropathic pain (Toth, 2014). Therefore, PGB has been used for treating painful symptoms in MS (Solaro et al., 2009), being classified as second-line pharmacological therapy for central neuropathic pain by the Brazilian Academy of Neurology (Oliveira et al., 2020). Besides its clinical effects, a single treatment with PGB has been associated with neuroprotective effect in animal models of epilepsy (André et al., 2003), spinal cord injury (Ha et al., 2011; Tedeschi et al., 2016; Erskine et al., 2019), and MS (Silva et al., 2014; Arima et al., 2015; Hundehege et al., 2018). Once some of these neuroprotective effects included modulation of glial cells, simultaneous assessment of the effect of PGB, and an immunomodulatory strategy such as DMF could optimize the beneficial response and reduce subsequent disease exacerbations. Therefore, the objective of the study was to investigate the glial response in EAE mice treated with a combination of DMF and PGB.

## Materials and methods

### Ethical approval, animal conditions, and study design

Experiments were carried out following the international guidelines and principles regulated by the National Council of Animal Experimentation for the care and use of animals (CONCEA, Brazil). After the ethics committee approval (4730-1/2017), the Multidisciplinary Center for Biological Research (CEMIB/UNICAMP, Campinas, SP, Brazil) supplied the 28 female C57BL/6J mice used in the present study. All animals were maintained in standard conditions in the animal house of the Laboratory of Nerve Regeneration on a 12/12-h light/dark cycle and were provided with food and water *ad libitum*. Eight- to nine-week-old animals were randomly assigned to the five experimental groups: naïve, EAE, EAE-DMF, EAE-PGB, and EAE-DMF + PGB. Pharmacological treatments were initiated with the beginning of clinical signs. Weight and clinical scores were collected from 0 to 28 days post-induction (dpi).

### Experimental autoimmune encephalomyelitis induction, clinical assessment, and drug treatments

Experimental autoimmune encephalomyelitis induction and the associated clinical signs evaluation were performed as previously described (Bernardes et al., 2013). Briefly, EAE mice received a subcutaneous injection with an emulsion containing 100 μg of MOG35–55 peptide (Proteimax Biotecnologia Ltda, São Paulo, Brazil) prepared with complete Freund’s adjuvant (CFA, F5881, Sigma-Aldrich, St. Louis, MO, United States), supplemented with 4 mg/ml Mycobacterium tuberculosis H37Ra (Difco Laboratories, Detroit, MI, United States). Bordetella pertussis toxin (300 ng/animal; P7208 Sigma-Aldrich, St. Louis, MO, United States) was resuspended in 100 μl of phosphate-buffered saline (PBS, pH 7.38) and injected intraperitoneally (i.p.) on the day of immunization and after 48 hours. Daily clinical evaluation was defined as follows: 0 – no clinical signs; 1 – tail paralysis (or loss of tail tone); 2 – tail paralysis and hind-limb weakness (visible paresis); 3 – one or two hind-limb paralyzes; and 4 – one or two hind-limb paralyzes with forelimb impairment. With the beginning of clinical signs, i.e., tail paralysis (11–12 dpi), and until 28 dpi, animals from the treated groups’ received daily gavage with DMF at a dose of 15 mg/kg (Linker et al., 2011; Bernardes and de Oliveira, 2018) and/or PGB at a dose of 30 mg/kg (Silva et al., 2014). Therefore, treatments were delivered within 17–18 days in total. Both drugs were diluted in 0.08% methylcellulose solution since this is the mandatory vehicle for DMF. The EAE group received daily vehicle gavage to match up with animal handling.

### Tissue processing and histopathology assessment

All tissue processing and histopathology assessments were performed as described elsewhere and the lumbar spinal cord was chosen because the tail and hindlimb impairments observed during disease correspond to the lumbar intumescence grey matter glial reactivity (Silva et al., 2014; Bernardes and de Oliveira, 2018). For that, at 28 dpi, all animals were anesthetized by a mixture of xylazine (80 mg/kg) and ketamine (400 mg/kg) and perfused with phosphate-buffered saline (PBS, pH 7.38) followed by buffered 4% paraformaldehyde solution. The lumbar spinal cord was collected, included in a cryopreservation medium (Tissue-Tek Optimal Cutting Temperature Compound, Sakura, Japan), and frozen with temperatures ranging from −30 to −35°C. The cryopreserved tissues were kept at −20°C until they were sectioned in cross-sections of 12 μm using a cryostat (MICROM, model HM505E). The slides were kept at −20°C until use.

For demyelination studies, the slides were acclimatized at room temperature for 2–3h, washed in distilled water for 1 min and dehydrated in 70% ethanol for 1 min. Afterward, the slides were incubated for 20 min in Sudan Black (Sigma-Aldrich, St. Louis, MO, United States) which was prepared at 0.7% proportion in 70% ethanol. Then, the slides were washed three times in 70% ethanol and rehydrated in distilled water for 30 s. For FluoroMyelin labeling, the acclimatized slides were washed in distilled water and incubated with FluoroMyelin for 30 min (ThermoFisher, F34652; 1:300), then washed three times. Finally, the slides were mounted and stored at −20°C. The stained slides were observed under a Leica DM 5500B light microscope and photographed with a high-sensitivity camera (DFC295, for brightfield images, and DFC345X for fluorescence images). Partial images of the spinal cord sections were obtained using a 10X objective and using Adobe Photoshop Elements 10 program, the full area of each spinal cord was assembled.

For the immunofluorescence studies, the slides were acclimatized at room temperature for 15 min, washed in phosphate buffer (PB 0.01M) three times for 5 min each, and incubated for 45 min with a blocking solution containing 3% fetal bovine serum (BSA) prepared in PB 0.01M. Slides used for nuclear factor (erythroid-derived 2)-like 2 (Nrf2) immunolabeling were previously incubated for 10 min in a permeabilization solution (0.25% Triton X-100 in PB 0.1M) and blocked with a 5% donkey serum solution. Then, anti-Iba1 (Ionized calcium-binding adaptor molecule 1; Rabbit, Wako 019-19741, 1:750) and anti-GFAP (glial fibrillary acidic protein; Rabbit, Abcam AB7260, 1:1500) were incubated on the slides for 3h. Anti-Nrf2 (Goat, Santa Cruz, sc-30915, 1:50) was incubated overnight at 4°C. Afterward, the slides were washed and incubated for 45 min with the fluorescent secondary antibody (Alexa Fluor 488; Anti-Rabbit IgG, Jackson, 1:500, and/or Alexa Fluor 594; Anti-Goat IgG, Jackson, 1:500). All the primary and secondary antibodies were prepared in a solution containing 100 mL of PB 0.01M, 1.5g of BSA, and 200 μl of Triton-X (Sigma-Aldrich, St. Louis, MO, United States). After that, the slides were washed in PB 0.01M three times for 5 min each, assembled, and reserved at −20°C. Immunolabeled slides were observed on a Leica DM 5500B fluorescence microscope coupled to a high-sensitivity camera (DFC 345FX, Leica, Germany). Images of ventral and dorsal horns were captured using a 20X objective.

The sectioning was performed in a series of 1:10 and five sections were placed per slide (total thickness of about 500–600 μm). For quantification, three representative images from the first to the fifth section in each specimen of every experimental group were selected. If necessary, another slide was used to maintain the distance sample. The mean integrated density of pixels (IDP) for immunofluorescence studies was calculated for each animal and then the mean ratio for each group ± standard error was established and normalized against the naive group. The demyelinating lesions were calculated as the percentage of the total myelin area. All quantifications were performed using ImageJ software (National Institutes of Health, United States).

For colocalization assessment, a set of multi-channel fluorescence images of Nrf2 and GFAP or Nrf2 and Iba-1 double labeling were processed using ImageJ (FIJI). Briefly, channels were separated, and single channels were submitted to threshold segmentation. After that, a one-to-one pixel matching analysis was carried out using the Coloc2 plugin. The Pearson’s correlation coefficient (Pearson’s *r*) was used for statistic quantification of colocalization.

### Statistical analysis

To investigate the interaction between disease and the treatment approaches, clinical score and body weight were analyzed by two-way ANOVA with the Newman-Keuls multiple comparisons test. Additionally, the whole time point curves and cumulative scores were compared by Mann-Whitney and unpaired t-tests. Histopathologic data were investigated using one-way ANOVA with the Newman-Keuls multiple comparisons test. All analyses were performed by GraphPad Prism 8.0.1 and the significance level was established at *P* < 0.05. All data were expressed as the mean ± SEM.

## Results

### The combination of dimethyl fumarate and pregabalin treatment attenuates the clinical severity of experimental autoimmune encephalomyelitis

**FIGURE 1:**
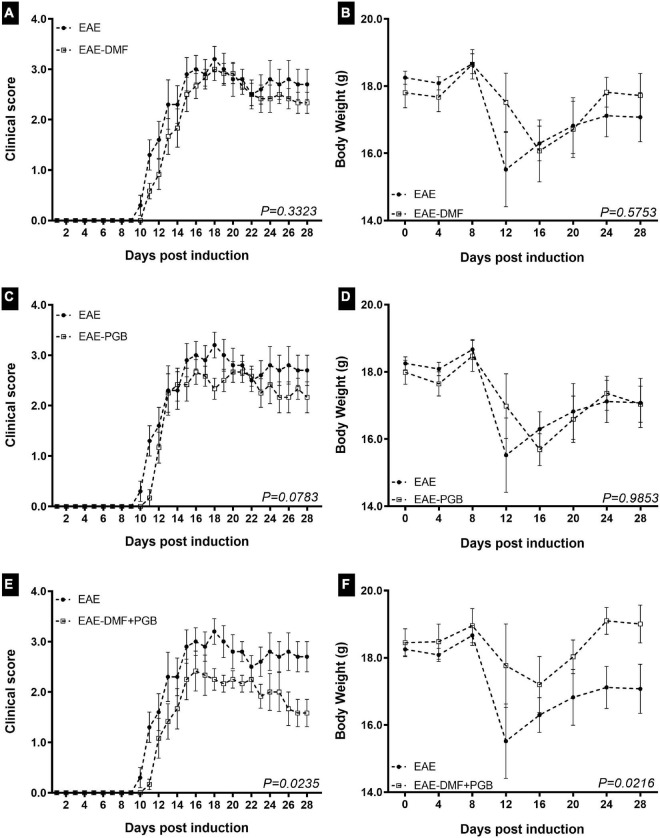
Clinical assessment and body weight of EAE mice treated with dimethyl fumarate (DMF), pregabalin (PGB), and the combination (DMF + PGB). **(A)** Clinical score of DMF group. There were disease (F_27, 252_ = 61.20 with *P* < 0.0001) and treatment effects (F_1, 252_ = 11.29 with *P* < 0.001) with no interaction between them (F_27, 252_ = 0.5201 with *P* = 0.9778). The cumulative clinical score was 47.20 ± 4.35 for EAE and 41.42 ± 3.15 for EAE-DMF (*P* > 0.05, *t*-test). **(B)** Body weight of DMF group. There was disease (F_7, 72_ = 3.186 with *P* = 0.0054) but not treatment effect (F_1, 72_ = 0.6525 with *P* = 0.4219) and no interaction between them (F_7, 72_ = 0.7794 with *P* = 0.6066). **(C)** Clinical score of PGB group. There were disease (F_27, 252_ = 53.74 with *P* < 0.0001) and treatment effects (F_1, 252_ = 16.48 with P < 0.001) with no interaction between them (F_27, 252_ = 0.8202 with P = 0.7240). The cumulative clinical score was 47.20 ± 4.35 for EAE and 39.92 ± 3.43 for EAE-PGB (*P* > 0.05, *t*-test). **(D)** Body weight of PGB group. There was disease (F_7, 72_ = 4.476 with *P* = 0.0004) but not treatment effect (F_1, 72_ = 0.0008846 with *P* = 0.9764) and no interaction between them (F_7, 72_ = 0.5644 with *P* = 0.7823). **(E)** Clinical score of DMF + PGB group. There were disease (F_27, 252_ = 40.34 with *P* < 0.0001) and treatment effects (F_1, 252_ = 53.62 with *P* < 0.001) with no interaction between them (F_27, 252_ = 1.302 with *P* = 0.1517). **(F)** Body weight of DMF + PGB group. There were disease (F_7, 72_ = 2.74 with *P* = 0.0140) and treatment effects (F_1, 72_ = 11.69 with *P* = 0.0010) with no interaction between them (F_7, 72_ = 0.7618 with *P* = 0.6210). The cumulative clinical score was 47.20 ± 4.35 for EAE and 33.17 ± 3.29 for EAE-DMF + PGB (*P* < 0.05, *t*-test). Both parameters were significantly decreased by the drug association (*P* – values of the whole curve are indicated, Mann-Whitney test). C57BL/6 mice from EAE (*n* = 5), EAE-DMF (*n* = 6), EAE-PGB (*n* = 6) and EAE-DMF + PGB (*n* = 6) were monitored by 28 days of disease. Treatments were initiated with the beginning of clinical scores.

To assess the effect of the treatments on the progression of the clinical signs of the disease model after the initiation of the treatments, EAE mice were monitored daily until 28 dpi (Figure 1). The two-way ANOVA demonstrated that the clinical score was highly associated with dpi and all treatment approaches used herein. However, there was no interaction between the two factors in all groups and no differences were observed by Newman-Keuls multiple comparisons test. Similarly, it has been observed as an important effect of the disease on the body weight progression, but only the combined DMF + PGB treatment significantly affected it. Likewise, there was no interaction between the two factors for body weight data and no differences were observed by Newman-Keuls multiple comparisons test. The F and the P values of the two-way ANOVA are demonstrated in the respective figure legend.

Noteworthy, the Mann-Whitney test of the whole time point curves demonstrated a significant difference between EAE and EAE-DMF + PGB groups for both clinical scores (Figure 1E) and body weight (Figure 1F), as well as the t-test of the cumulative scores (see figure legend). Considering the days most EAE animals reached the hind limb paralysis (13 to 28 dpi), a median clinical score of 3 was reached in 9, 7, 7, and 2 days for EAE, EAE-DMF, EAE-PGB, and EAE-DMF + PGB groups, respectively. Similarly, a median clinical score of 2.5 was reached in 7, 3, 4, and 2 days for EAE, EAE-DMF, EAE-PGB, and EAE-DMF + PGB groups, respectively. On the other hand, a median clinical score of 2 was reached in 6, 5, and 7 days for EAE-DMF, EAE-PGB, and EAE-DMF + PGB groups, respectively, while the median clinical score of 1.5 was only observed in the EAE-DMF + PGB group for five days. Taken together, these data suggest a stronger attenuation of the clinical presentation of EAE by the association of DMF and PGB than the single approaches themselves.

### The combination of dimethyl fumarate and pregabalin treatment attenuates demyelination in experimental autoimmune encephalomyelitis mice

**FIGURE 2:**
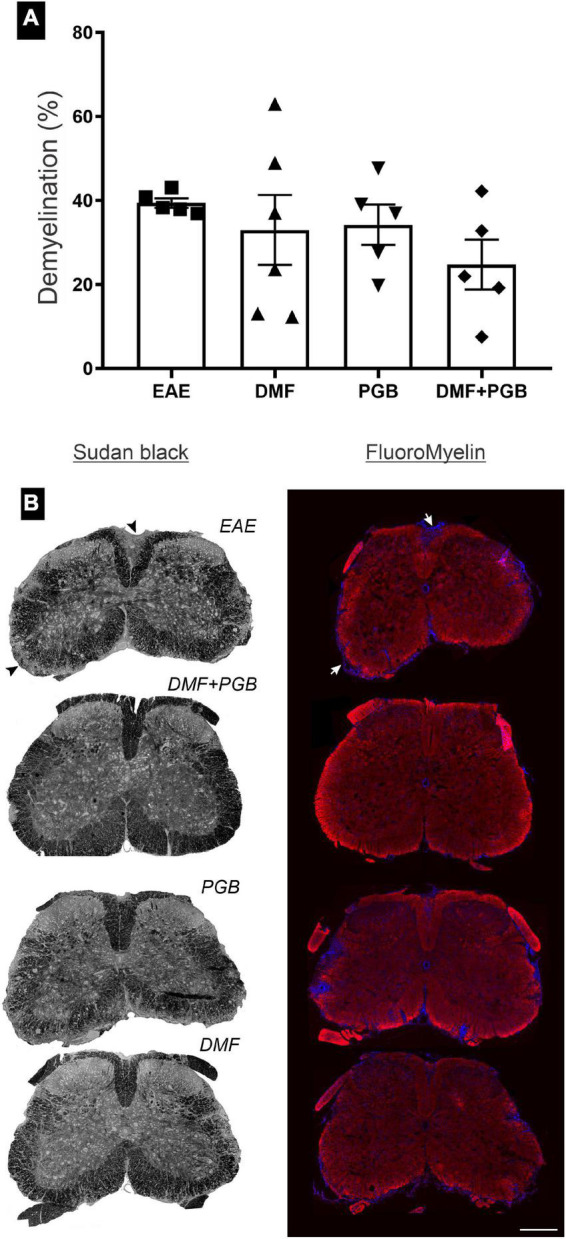
Spinal cord demyelinated areas at 28 dpi of EAE mice treated with dimethyl fumarate (DMF), pregabalin (PGB), and the association (DMF + PGB). **(A)** Quantification of the demyelinated areas from the Sudan Black labeling. The one-way ANOVA of the volume of demyelination of the four groups disclosed no significant differences (F3, 17 = 0.9561 with *P* = 0.4359). **(B)** Representative images of lumbar spinal cord sections stained with Sudan Black and FluoroMyelin. Scale bar = 300 μm.

In Figure 2B, it is possible to observe the representative images of lumbar spinal cord sections stained with Sudan Black and fluoromyelin with attenuated demyelination in the EAE animals treated with the combined drug treatment (DMF + PGB). The quantification of the volume of demyelination, obtained from the Sudan Black labeling, data (Figure 2A) of the four groups, disclosed no significant differences. However, the mean ± SEM was 39.39 ± 1.13%, 32.97 ± 8.34%, 34.21 ± 4.82%, and 24.72 ± 5.94% for EAE, EAE-DMF, EAE-PGB, and EAE-DMF + PGB groups, respectively, suggesting an attenuation of the demyelination by the combination of DMF and PGB.

### The combination of dimethyl fumarate and pregabalin treatment attenuates glial reactivity in experimental autoimmune encephalomyelitis mice and upregulates the expression of Nrf2 mostly in astrocytes

**FIGURE 3:**
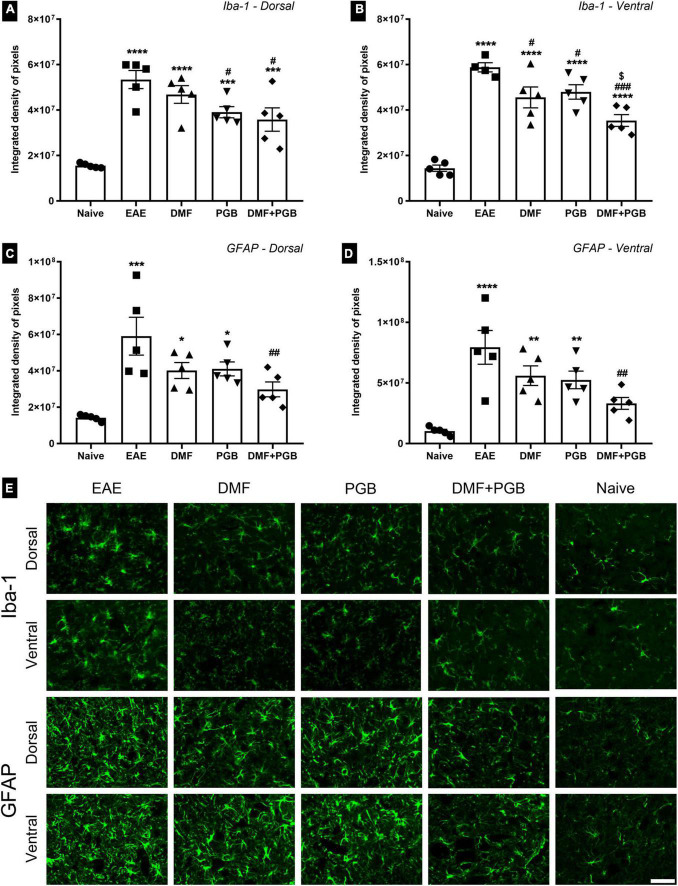
Spinal cord glial reactivity at 28 dpi of EAE mice treated with dimethyl fumarate (DMF), pregabalin (PGB), and the combination (DMF + PGB). **(A)** Quantification of the integrated density of pixels (IDP) of pictures immunolabeled with anti-Iba1 for microglial cells of the dorsal root entry zone. The one-way ANOVA of IDP data demonstrated the significant effects of the treatments used herein (F_4, 20_ = 16.21 with *P* < 0.0001). ****P < 0.0001 and ***P < 0.001 compared to the naïve group; ^#^*P* < 0.05 compared to the EAE group. **(B)** Quantification of IDP of pictures immunolabeled with anti-Iba1 for microglial cells of the ventral horn. The one-way ANOVA of IDP data demonstrated the significant effects of the treatments used herein (F_4, 19_ = 29.57 with *P* < 0.0001). ****P < 0.0001 compared to the naïve group; ^###^*P* < 0.0001 and ^#^*P* < 0.05 compared to the EAE group; ^[*$*]^*P* < 0.05 compared to the DMF and PGB groups. **(C)** Quantification of the IDP of pictures immunolabeled with anti-GFAP for astrocytes of the superficial dorsal horn (the one-way ANOVA demonstrated a strong effect of the treatments used herein for both dorsal with F_4, 20_ = 8.49 and *P* = 0.0004). ***P < 0.0001 and *P < 0.05 compared to the naïve group; ^##^*P* < 0.01 compared to the EAE group. **(D)** Quantification of IDP in astrocytes of the ventral horn immunolabeled with anti-GFAP (the one-way ANOVA demonstrated a strong effect of the treatments used herein for both dorsal with F_4, 20_ = 9.96 and *P* = 0.0001). ****P < 0.0001 and **P < 0.01 compared to the naïve group; ^##^P < 0.01 compared to the EAE group. **(E)** Representative images of spinal cord regions immunolabeled with anti-Iba1 and anti-GFAP of C57BL/6 mice from the EAE, EAE-DMF, EAE-PGB, EAE-DMF + PGB and naïve groups. Scale bar: 50 μm.

We evaluated the response of microglia (Iba-1 marker) and astrocytes (GFAP marker) by immunofluorescence in the gray matter of the spinal cord of EAE mice treated with DMF, PGB, and the combined drug treatments (DMF + PGB) at 28 dpi (Figure 3). Regarding anti-Iba1 of the dorsal root entry zone, all four EAE groups demonstrated increased IDP value compared to naïve and the EAE-PGB and EAE-DMF + PGB groups demonstrated decreased IDP value compared to the EAE group (Figures 3A,E). In the ventral horn, a similar effect was observed with increased IDP value of all four EAE groups compared to naïve. However, all the treatment approaches were efficient in decreasing IDP value compared to the EAE group, and the combined drug treatment (DMF + PGB) additionally decreased the IDP value in comparison with the single treatment approaches (Figures 3B,E).

Regarding the IDP data of pictures immunolabeled with anti-GFAP, only EAE and the single-treated groups (EAE-DMF and EAE-PGB) demonstrated increased IDP values compared to naïve. Besides, the combined drug treatment (DMF + PGB) decreased the IDP value in comparison with the EAE group for both dorsal (Figures 3C,E) and ventral (Figures 3D,E) horns. Taken together, these data suggest a strong attenuation of glial response in EAE mice treated with the combination of DMF and PGB.

**FIGURE 4:**
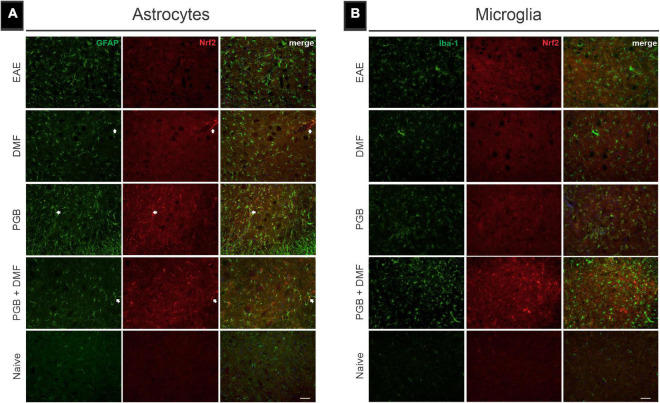
Nuclear factor (erythroid-derived 2)-like 2 (Nrf2) immunolabeling in combination with glial markers in EAE-induced mice treated with dimethyl fumarate (DMF), pregabalin (PGB), and the combination DMF + PGB. **(A)** Representative images of transverse spinal cord sections from EAE, DMF, PGB, DMF + PGB, and naïve groups double-labeled with glial fibrillary acidic protein (GFAP, green; astrocyte marker), and Nrf2 (red). White arrows indicate the preferential localization of Nrf2 expression in perivascular astrocytes. **(B)** Representative images of transverse spinal cord sections double-labeled for microglia/macrophage (Iba-1, green), and Nrf2 (red). Scale bar: 50 μm.

In addition to the glial reactivity mitigation, we studied the possibility that DMF and PGB treatment influenced the expression of Nrf2. For that, double labelings of GFAP and Iba-1 were performed with an anti-Nrf-2 antibody (Figure 4). The results indicate that PGB alone or associated with DMF upregulates Nrf-2 throughout the spinal cord. Partial colocalization with GFAP labeling indicates that astrocytes are responsible for part of the Nrf-2 upregulation seen herein (Figure 4A) (Naïve = 0.07 ± 0.03; EAE = 0.20 ± 0.03; DMF = 0.23 ± 0.04; PGB = 0.32 ± 0.07; DMF + PGB = 0.29 ± 0.08; correlative analysis of GFAP and Nrf2 using Pearson’s r, Mean ± SD). Iba-1, on the other hand, did not show colocalization with Nrf-2, indicating that microglial cells are not the source of such immunoreactivity (Naïve = 0.06 ± 0.03; EAE = 0.08 ± 0.03; DMF = 0.04 ± 0.02; PGB = 0.08 ± 0.04; DMF + PGB = 0.05 ± 0.01; correlative analysis of Iba-1 and Nrf2 using Pearson’s r, Mean ± SD) (Figure 4B).

## Discussion

In the present study, treatment with 15 mg/kg/day of DMF or 30 mg/kg/day of PGB alone administered by gavage from the onset of disease (11-12 dpi) to 28 dpi was not enough to positively modulate the clinical presentation of MOG-induced EAE in C57BL/6 mice. DMF administrated in a similar dosage two times a day (Linker et al., 2011) or with a ten-fold dosage (150 mg/kg) administered one time a day (Parodi et al., 2015) was associated with clinical score amelioration in the same EAE model. Although reducing DMF dosage may be reasonable because of some collateral effects (Gafson et al., 2019), it may be insufficient to reach beneficial clinical improvements in animal models. However, one study applying 15 mg/kg of DMF two times a day did not demonstrate an EAE clinical improvement as well, and the authors raised the suggestion that DMF may be more effective when the clinical score is limited to gait ataxia than when hind limb paralysis is observed (das Neves et al., 2020). The same suggestion could be addressed to PGB-treated EAE animals. For example, 30 mg/kg of PGB administered i.p. one time a day resulted in an amelioration of the disease score of MOG-induced EAE in C57BL/6 mice that reached only paraparesis (Hundehege et al., 2018). Most of our non-treated EAE mice had reached hind limb paralysis at 13 dpi, demonstrating that the disease presentation in our study was severe and could be suited to such justification. Some authors have also observed that DMF alone did not promote clinical attenuation in three different EAE models (de Bruin et al., 2016) or Lewis rats’ model in either situation: when administered two times a day at a dose of 15 mg/kg (Kasarełło et al., 2017) or even in a high dosage such as 120 mg/kg/day (Pouzol et al., 2019). Similarly, PGB at 30 mg/kg/day promoted a delayed course of the disease without an overt attenuation in a Lewis rat EAE model (Silva et al., 2014). Importantly, some of these studies observed the necessity of a combination of DMF and other neuroprotective approaches as well as a combination of PGB and another immunomodulatory strategy to reach a more effective clinical mitigation. Additionally, the combination of these pharmacotherapies may eventually be necessary for the parallel management of the disease itself and the related neuropathic symptoms. Thus, our data suggest a stronger attenuation of the clinical presentation of EAE by the combination of DMF and PGB than the single approaches alone.

Noteworthy, decreased demyelination in the spinal cord was associated with clinical score improvement mostly in EAE animals that reached a mild clinical presentation after both single DMF (Linker et al., 2011) and single PGB (Hundehege et al., 2018) treatments. Decreased demyelination lesions in the hippocampus of EAE mice treated with DMF were associated with improved cognitive performance even though the clinical score was not affected by the treatment (das Neves et al., 2020). Taken together, these data suggest that to reach significant effects from these pharmacological approaches on demyelinating lesions a mild demyelinating area must be observed. Therefore, our dosage scheme of the single treatment did not produce myelin preservation, while the association has had only a small effect, possibly because of the severe clinical and demyelination presentation seen herein. However, we cannot dispute the fact that the clinical attenuation observed in our study by the combined drugs was associated with a slight attenuation of demyelination volume in the spinal cord, i.e., ≈15% of reduction when EAE and EAE-DMF + PGB groups are compared. Most importantly, a further reduction of glial reactivity in the gray matter of the lumbar spinal cord was observed by the drug combination used in the present study.

Microglia reactivity has been associated with the exacerbation of the disease in different EAE models (Linker et al., 2011; Silva et al., 2014; Bernardes and de Oliveira, 2018; das Neves et al., 2020). However, even with no clear effect on clinical score presentation and demyelination volume, both single DMF and PGB reduced Iba-1 immunolabeling in the ventral horn while PGB alone reduced Iba-1 immunolabeling also in the dorsal horn of the lumbar spinal cord. Such pharmacological strategies have been associated with some contradictory results regarding microglia and associated clinical score and/or behavioral presentation in EAE models. Better performance on the Catwalk gait analysis method was paralleled by increased Iba-1 immunolabeling in the gray matter of the lumbar spinal cord of animals that received 15 mg/kg/day of DMF and performed five weeks of exercise before the EAE induction (Bernardes and de Oliveira, 2018). Similarly, cognitive improvement of single DMF treatment was associated with increased Iba-1 marker in the fimbria of EAE mice (das Neves et al., 2020). In addition, clinical score amelioration was associated with no effect in Mac-3 positive macrophages and microglia after DMF treatment on 74th dpi of EAE mice (Linker et al., 2011). Likewise, in EAE mice, microglial cells were unaffected by PGB even with clinical score attenuation (Hundehege et al., 2018). Besides, PGB treatment promoted reduced Iba-1 immunolabeling in the ventral horn of the lumbar spinal cord of Lewis rats EAE model during the exacerbation of the disease even with no overt clinical score attenuation (Silva et al., 2014). Taken together, these data suggest that DMF and PGB alone may have positive effects on microglia that are not always connected to a better clinical or behavioral presentation.

Importantly, the cognitive improvement of EAE mice treated with DMF was associated with reduced astrocytic reactivity in the fimbria (das Neves et al., 2020). Such as microglia, PGB single treatment promoted reduced GFAP immunolabeling in the ventral horn of the lumbar spinal cord of Lewis rats EAE (Silva et al., 2014). Herein, only the combination of DMF and PGB could reduce astrocyte reactivity in both the dorsal and ventral horn of the spinal cord of EAE mice which may be related to the apparent clinical score amelioration. Noteworthy, reduced GFAP has been associated with increased reactivity of synaptophysin by both DMF (Bernardes and de Oliveira, 2018) and PGB (Silva et al., 2014).

In neurons, oligodendrocytes, and astrocytes, the DMF-associated mechanism of action has been suggested to be the activation of the nuclear factor (erythroid-derived 2)-related factor-2 (Nrf2) that promotes increased expression of genes related to the oxidative stress response pathway (Linker et al., 2011). Our results are in line with that possibility, since we observed upregulation of Nrf2 immunolabeling, mostly in colocalization with GFAP, after treatment with DMF and the combination of DMF and PGB. Interestingly, Nrf-2 immunoreactivity was upregulated after PGB administration. This indicates that PGB also influences the expression of Nrf-2, which is in line with a previous work that investigated contextual memory deficits in streptozotocin-induced mice (Salat et al., 2016).

In microglia, it has been demonstrated that the DMF metabolite, monomethyl fumarate, binds to hydroxycarboxylic acid receptor 2 (HCAR2) which is coupled to Gq protein that increases intracellular calcium that activates a downstream pathway causing inhibition of NF-κB, which controls the expression of multiple inflammatory cytokines (Parodi et al., 2015). It has been demonstrated a reduced synthesis of pro-inflammatory cytokines such as TNF-α, IL-1β, and IL-6 and reduced oxidative profile in LPS-activated microglia and astrocytes by DMF administration in cultured cells (Wilms et al., 2010; Lin et al., 2011; Parodi et al., 2015; Michell-Robinson et al., 2016). Therefore, the antioxidative pathway in astrocytes and increased calcium in microglia are suggested as a two-way effect of DMF in microglia and astrocyte by DMF.

Alternatively, the PGB-associated mechanism of action is the inhibition of calcium channels predominantly on excitatory glutamatergic neurons (André et al., 2003; Tedeschi et al., 2016; Hundehege et al., 2018). Administration of PGB for seven days (46 mg/kg; three times/day) was associated with reduced synaptic transmission and axon regeneration after spinal cord injury (Tedeschi et al., 2016). In a pilocarpine model of epilepsy in rats, PGB promoted longer latency of spontaneous seizures and neuroprotection in layer II of the piriform cortex (André et al., 2003). In EAE mice, PGB has been associated with reduced long-term potentiation in hippocampal brain slices indicating an impact on mechanisms of learning and memory (Hundehege et al., 2018). Importantly, the neuroprotective effects in different animal models have been associated with decreased astrocyte (André et al., 2003; Hundehege et al., 2018) and microglia reactivity (Ha et al., 2011). There is some evidence suggesting that PGB reduces the cytotoxicity mediated by calcium and thereby the neuronal damage in the EAE model (Hundehege et al., 2018).

Overall, it is possible that the combination used herein caused increased intracellular calcium in microglia (DMF effect) but decreased intracellular calcium in neurons (PGB effect). The differential effect on microglia and neurons putatively prevented the astrocyte from activation and maintained these cells at a non-polarized profile. Taken together, these data suggest that the minimal effect observed with single DMF and PGB on glial cells has been strengthened when these two pharmacotherapies were combined. The mechanism of action may be related to a differential calcium intracellular metabolism associated with an antioxidative response, culminating in an optimized attenuation of glial cells that paralleled the reduction of the clinical score. However, further investigations are necessary to confirm such a hypothesis.

## Data availability statement

The raw data supporting the conclusions of this article will be made available by the authors, without undue reservation.

## Ethics statement

The animal study was reviewed and approved by Ethics Committee on the Use of Animals/University of Campinas- 4730-1/2017.

## Author contributions

AH, DB, LC, and AO contributed to the conception and design and wrote the manuscript. AH, DB, and LC contributed to the acquisition, analysis, and editing of data. AO provided the supervision, curated data, and revised the manuscript. All authors read and approved the final manuscript.

## References

[B1] Aharoni R. Eilam R. Arnon R. (2021). Astrocytes in multiple sclerosis—essential constituents with diverse multifaceted functions. *Internat. J. Mole. Sci.* 22:22115904. 10.3390/ijms22115904 34072790 PMC8198285

[B2] André V. Rigoulot M.-A. Koning E. Ferrandon A. Nehlig A. (2003). Long-term pregabalin treatment protects basal cortices and delays the occurrence of spontaneous seizures in the lithium-pilocarpine model in the rat. *Epilepsia* 44 893–903. 10.1046/j.1528-1157.2003.61802.x 12823571

[B3] Arima Y. Kamimura D. Atsumi T. Harada M. Kawamoto T. Nishikawa N. (2015). A pain-mediated neural signal induces relapse in murine autoimmune encephalomyelitis, a multiple sclerosis model. *Elife* 4 1–23. 10.7554/eLife.08733.001 PMC4530187 26193120

[B4] Bernardes D. de Oliveira A. L. R. (2018). Regular exercise modifies histopathological outcomes of pharmacological treatment in experimental autoimmune encephalomyelitis. *Front. Neurol.* 9:950. 10.3389/fneur.2018.00950 30524355 PMC6256135

[B5] Bernardes D. Oliveira-Lima O. C. da Silva T. V. Faraco C. C. F. Leite H. R. Juliano M. A. (2013). Differential brain and spinal cord cytokine and BDNF levels in experimental autoimmune encephalomyelitis are modulated by prior and regular exercise. *J. Neuroimmunol.* 264 24–34. 10.1016/j.jneuroim.2013.08.014 24054000

[B6] Correale J. Marrodan M. Ysrraelit M. C. (2019). Mechanisms of neurodegeneration and axonal dysfunction in progressive multiple sclerosis. *Biomedicines* 7:7010014. 10.3390/biomedicines7010014 30791637 PMC6466454

[B7] Dargahi N. Katsara M. Tselios T. Androutsou M. E. de Courten M. Matsoukas J. (2017). Multiple sclerosis: Immunopathology and treatment update. *Brain Sci.* 7:78. 10.3390/brainsci7070078 28686222 PMC5532591

[B8] das Neves S. P. Santos G. Barros C. Pereira D. R. Ferreira R. Mota C. (2020). Enhanced cognitive performance in experimental autoimmune encephalomyelitis mice treated with dimethyl fumarate after the appearance of disease symptoms. *J. Neuroimmunol.* 340:577163. 10.1016/j.jneuroim.2020.577163 31982706

[B9] de Bruin N. M. W. J. Schmitz K. Schiffmann S. Tafferner N. Schmidt M. Jordan H. (2016). Multiple rodent models and behavioral measures reveal unexpected responses to FTY720 and DMF in experimental autoimmune encephalomyelitis. *Behav. Brain Res.* 300 160–174. 10.1016/j.bbr.2015.12.006 26692368

[B10] Dendrou C. A. Fugger L. Friese M. A. (2015). Immunopathology of multiple sclerosis. *Nat. Rev. Immunol.* 15 545–558. 10.1038/nri3871 26250739

[B11] Erskine E. L. K. S. Smaila B. D. Plunet W. Liu J. Raffaele E. E. Tetzlaff W. (2019). Skilled reaching deterioration contralateral to cervical hemicontusion in rats is reversed by pregabalin treatment conditional upon its early administration. *Pain Rep.* 4:749. 10.1097/PR9.0000000000000749 31583362 PMC6749902

[B12] Gafson A. R. Savva C. Thorne T. David M. Gomez-Romero M. Lewis M. R. (2019). Breaking the cycle: reversal of flux in the tricarboxylic acid cycle by dimethyl fumarate. *Neurology* 6:562. 10.1212/NXI.0000000000000562 31086805 PMC6481230

[B13] Gholamzad M. Ebtekar M. Ardestani M. S. Azimi M. Mahmodi Z. Mousavi M. J. (2019). A comprehensive review on the treatment approaches of multiple sclerosis: currently and in the future. *Inflamm. Res.* 68 25–38. 10.1007/s00011-018-1185-0 30178100

[B14] Greenhalgh A. D. David S. Bennett F. C. (2020). Immune cell regulation of glia during CNS injury and disease. *Nat. Rev. Neurosci.* 21 139–152. 10.1038/s41583-020-0263-9 32042145

[B15] Ha K. Y. Carragee E. Cheng I. Kwon S. E. Kim Y. H. (2011). Pregabalin as a neuroprotector after spinal cord injury in rats: Biochemical analysis and effect on glial cells. *J. Kor. Med. Sci.* 26 404–411. 10.3346/jkms.2011.26.3.404 21394310 PMC3051089

[B16] Hundehege P. Fernandez-Orth J. Römer P. Ruck T. Müntefering T. Eichler S. (2018). Targeting voltage-dependent calcium channels with pregabalin exerts a direct neuroprotective effect in an animal model of multiple sclerosis. *NeuroSignals* 26 77–93. 10.1159/000495425 30481775

[B17] Kasarełło K. Jesion A. Tyszkowska K. Matusik K. Czarzasta K. Wrzesień R. (2017). Effect of dimethyl fumarate on heme oxygenase-1 expression in experimental allergic encephalomyelitis in rats. *Folia Neuropathol.* 55 325–332. 10.5114/fn.2017.72394 29363907

[B18] Lian G. Gnanaprakasam R. Wang T. Wu R. Chen X. Liu L. (2018). Glutathione de novo synthesis but not recycling process coordinates with glutamine catabolism to control redox homeostasis and directs murine T cell differentiation. *Elife* 7 1–28. 10.7554/eLife.36158 PMC6152796 30198844

[B19] Lin S. X. Lisi L. Russo C. dello Polak P. E. Sharp A. (2011). The anti-inflammatory effects of dimethyl fumarate in astrocytes involve glutathione and haem oxygenase-1. *ASN Neuro* 3:AN20100033. 10.1042/AN20100033 21382015 PMC3072764

[B20] Linker R. A. Lee D. H. Ryan S. van Dam A. M. Conrad R. Bista P. (2011). Fumaric acid esters exert neuroprotective effects in neuroinflammation *via* activation of the Nrf2 antioxidant pathway. *Brain* 134 678–692. 10.1093/brain/awq386 21354971

[B21] Michell-Robinson M. A. Moore C. S. Healy L. M. Osso L. A. Zorko N. Grouza V. (2016). Effects of fumarates on circulating and CNS myeloid cells in multiple sclerosis. *Ann. Clin. Transl. Neurol.* 3 27–41. 10.1002/acn3.270 26783548 PMC4704479

[B22] Mills E. A. Ogrodnik M. A. Plave A. Mao-Draayer Y. (2018). Emerging understanding of the mechanism of action for dimethyl fumarate in the treatment of multiple sclerosis. *Front. Neurol.* 9:0005. 10.3389/fneur.2018.00005 29410647 PMC5787128

[B23] Moharregh-Khiabani D. Linker R. A. Gold R. Stangel M. (2009). Fumaric acid and its esters: an emerging treatment for multiple sclerosis. *Curr. Neuropharmacol.* 7 60–64. 19721818 10.2174/157015909787602788 PMC2724664

[B24] MSIF (2020). The Multiple Sclerosis International Federation, Atlas of MS, 3rd Edition. Available online at: www.atlasofms.org (accessed March 14, 2022).

[B25] Oliveira R. A. A. de Baptista A. F. Sá K. N. Barbosa L. M. Nascimento O. J. M. (2020). Pharmacological treatment of central neuropathic pain: consensus of the brazilian academy of neurology. *Arquivos de Neuro-Psiquiatria* 78:166. 10.1590/0004-282x20200166 33331468

[B26] Parodi B. Rossi S. Morando S. Cordano C. Bragoni A. Motta C. (2015). Fumarates modulate microglia activation through a novel HCAR2 signaling pathway and rescue synaptic dysregulation in inflamed CNS. *Acta Neuropathol.* 130 279–295. 10.1007/s00401-015-1422-3 25920452 PMC4503882

[B27] Pouzol L. Piali L. Bernard C. Martinic M. M. Steiner B. Clozel M. (2019). Therapeutic potential of ponesimod alone and in combination with dimethyl fumarate in experimental models of multiple sclerosis. *Innov. Clin. Neurosci.* 16 22–30. 31214480 PMC6538399

[B28] Ransohoff R. M. Hafler D. A. Lucchinetti C. F. (2015). Multiple sclerosis – a quiet revolution. *Nat. Rev. Neurol.* 11 134–142. 10.1038/nrneurol.2015.14 25686758 PMC4556342

[B29] Salat K. Gdula-Argasinska J. Malikowska N. Podkowa A. Lipkowska A. Librowski T. (2016). Effect of pregabalin on contextual memory deficits and inflammatory state-related protein expression in streptozotocin-induced diabetic mice. *Naunyn Schmiedebergs Arch Pharmacol* 389 613–623. 10.1007/s00210-016-1230-x 26984821 PMC4866991

[B30] Salter A. Lancia S. Cutter G. Fox R. J. Marrie R. A. Mendoza J. P. (2021). Characterizing long-term disability progression and employment in narcoms registry participants with multiple sclerosis taking dimethyl fumarate. *Internat. J. MS Care* 23 239–244. 10.7224/1537-2073.2020-109 35035294 PMC8745234

[B31] Silva G. A. A. Pradella F. Moraes A. Farias A. dos Santos L. M. B. de Oliveira A. L. R. (2014). Impact of pregabalin treatment on synaptic plasticity and glial reactivity during the course of experimental autoimmune encephalomyelitis. *Brain Behav.* 4 925–935. 10.1002/brb3.276 25365796 PMC4178248

[B32] Solaro C. Boehmker M. Tanganelli P. (2009). Pregabalin for treating paroxysmal painful symptoms in multiple sclerosis: a pilot study. *J. Neurol.* 256 1773–1774. 10.1007/s00415-009-5203-6 19579001

[B33] Tedeschi A. Dupraz S. Laskowski C. J. Xue J. Ulas T. Beyer M. (2016). The calcium channel subunit alpha2delta2 suppresses axon regeneration in the adult CNS. *Neuron* 92 419–434. 10.1016/j.neuron.2016.09.026 27720483

[B34] Toth C. (2014). Pregabalin: latest safety evidence and clinical implications for the management of neuropathic pain. *Therap. Adv. Drug Saf.* 5 38–56. 10.1177/2042098613505614 25083261 PMC4110876

[B35] Wilms H. Sievers J. Rickert U. Rostami-Yazdi M. Mrowietz U. Lucius R. (2010). Dimethylfumarate inhibits microglial and astrocytic inflammation by suppressing the synthesis of nitric oxide, IL-1β, TNF-α and IL-6 in an *in-vitro* model of brain inflammation. *J. Neuroinflam.* 7:30. 10.1186/1742-2094-7-30 20482831 PMC2880998

